# Zeolite-Y-Loaded Chitosan Nanoparticles as Endodontic Antimicrobial Agent: An *In vitro* Study

**DOI:** 10.1055/s-0045-1802947

**Published:** 2025-04-23

**Authors:** Amir Isam Omer Ibrahim, Desigar Moodley, Ernest Maboza, Annette Olivier, Leslie Petrik

**Affiliations:** 1 Department of Restorative Dental Sciences, College of Dentistry, King Faisal University, Al-Ahsa, Saudi Arabia; 2 Department of Restorative Dentistry, Faculty of Dentistry, University of the Western Cape, Cape Town, South Africa; 3 Edelweiss Dentistry Products Gmbh, Wolfurt, Austria; 4 WHO Collaborating Centre for Oral Health, Faculty of Dentistry, University of the Western Cape, Cape Town, South Africa; 5 Department of Chemistry, Faculty of Natural Sciences, University of the Western Cape, Cape Town, South Africa

**Keywords:** chitosan nanoparticles, intracanal medicament, antimicrobial, zeolite, cytotoxicity

## Abstract

**Objectives:**

The objectives were to synthesize a bioactive nanocomposite as an endodontic antimicrobial agent by loading previously synthesized electrosprayed chitosan nanoparticles (Ch-Np) into Zeolite-Y as a carrier and compare its antimicrobial activity against two endodontic pathogens using the agar diffusion test. Additionally, the effect of tissue inhibitors (dentin powder and serum albumin) on the antimicrobial activity of the Ch-Np-Zeolite nanocomposite was studied. Finally, the possible cytotoxicity of the novel nanocomposite against Balb/c 3T3 mouse fibroblast cells was evaluated.

**Materials and Methods:**

A concentration of 3% (w/v) electrosprayed Ch-Np was mixed with Zeolite-Y in a concentration of 53.3 (w/v) and characterized using high-resolution scanning electron microscopy (HR-SEM) and energy-dispersive X-ray spectroscopy (EDS) analysis. The antimicrobial activity was evaluated against *Streptococcus mutans* and *Enterococcus faecalis* using the agar diffusion test, and the time-kill test was performed using the broth microdilution technique in the presence of tissue inhibitors. The cytotoxicity was evaluated against 3T3 mouse fibroblast cells using the standard 3-(4,5-dimethylthiazol-2-yl)-2,5-diphenyltetrazolium bromide assay.

**Statistical Analysis:**

The difference between the antimicrobial activity of Ch-Np-Zeolite nanocomposite against *S. mutans* and *E. faecalis* was analyzed using the Mann–Whitney's *U* test. The effect of tissue inhibitors on the antimicrobial activity of Ch-Np-Zeolite nanocomposite was analyzed by comparing the mean of log colony-forming unit per milliliter over time. For the cytotoxicity assay, a statistically significant difference between each group and their control was made using a *t* -test with a probability value of *p* ≤ 0.05, considered a significant difference.

**Results:**

HR-SEM of the dried paste-like mixture Ch-Np-Zeolite revealed the typical crystal habit of the supporting zeolite, and EDS analysis confirmed that the zeolite parent material retained its elemental composition after loading with Ch-Np. The antimicrobial activity of Ch-Np-Zeolite was demonstrated by the mean diameter inhibition zones of 9.57 and 7.85 mm for *S. mutans* and *E. faecalis* , respectively. *Streptococcus mutans* and *E. faecalis* were completely eradicated in the presence of tissue inhibitors. The Ch-Np-Zeolite nanocomposite significantly promoted the growth of 3T3 fibroblast cells ( *p* < 0.05), supporting its lack of cytotoxicity.

**Conclusion:**

Zeolite-Y-loaded Ch-Np nanocomposite shows promising antimicrobial activity while maintaining its biocompatibility even in the presence of tissue inhibitors.

## Introduction

Traditional treatment modalities for endodontic diseases aim to eliminate the microbial load inside the root canal system by mechanically removing infected dentin in conjunction with the use of antimicrobial agents, such as root canal irrigants and intracanal medicaments, as well as to seal the root canal system three-dimensionally and thus promote healing. 1

Intracanal medicaments are essential—especially in multivisit endodontic treatment and in cases of endodontic necrosis—because they allow the elimination of retained endodontic pathogens that were not removed during mechanical root canal preparation or root canal irrigation. 2 3 Several intracanal medicaments are available; however, some intracanal medicaments' antimicrobial efficacy can be compromised due to their interaction with the inorganic component of the dentine structure and the organic component of the root canal system. 4 In addition, the emergence of some resistant pathogens further affects the antimicrobial efficacy of the commonly used intracanal medicament. 3 5 6 7 Moreover, various degrees of inflammatory reaction in the periapical tissues have been reported when in contact with some intracanal medicaments. 8 9 10

Chitosan nanoparticles (Ch-Np), synthesized using the electrospraying technique, demonstrated antimicrobial activity against resistant endodontic pathogens in planktonic and biofilm states while maintaining cytotoxicity. 11 Hence, to be used as an intracanal medicament, it needs a carrier that does not affect its antimicrobial properties, even in the presence of tissue inhibitors, such as dentine powder and serum albumin. Zeolites are hydrated sodium aluminosilicate microporous crystalline materials (Na _12_ [(−SiO _2_ ) _12_ (AlO _2_ ) _12_ ]·27H _2_ O). 12 13 Their structure is based on a three-dimensional network composed of SiO _4_ and AlO _4_ tetrahedra that are corner-shared to form porous materials with various pore sizes. 14 Several nanoparticles can be admixed with zeolites, which allow the controlled distribution of nanoparticles. 15 In endodontics, Correia et al 16 developed bioactive glass nanoparticles that incorporated magnesium oxide and copper oxide to serve as an intracanal medicament. The developed nanocomposite showed promising antimicrobial activity against selected endodontic pathogens in a planktonic state without evaluating its biocompatibility.

Therefore, the aims of this study were to first design a novel Ch-Np-Zeolite nanocomposite as an endodontic antimicrobial agent by loading previously characterized electrosprayed Ch-Np onto Zeolite-Y. Second, it aimed to evaluate and compare the antimicrobial activity of the Ch-Np-Zeolite nanocomposite against two endodontic pathogens using the agar diffusion test. The next aim was to evaluate the effect of tissue inhibitors on the antimicrobial activity of the Ch-Np-Zeolite nanocomposite. Finally, the study aimed to evaluate the cytotoxicity of the novel nanocomposite against 3T3 mouse fibroblast cells.

## Materials and Methods

Before starting this study, ethical approval was obtained from the Senate Research Committee, University of the Western Cape, South Africa (BM/16/5/2). The methodology is illustrated in the flowchart shown in Supplementary Fig. S1 (available in the online version only).

### Synthesis and Characterization of Ch-Np-Zeolite Nanocomposite

To synthesize the novel Ch-Np-Zeolite nanocomposite, 90 mg of Ch-Np powder was dissolved in 3 mL of deionized water. The mixture was stirred at 1,000 rpm for 1 hour using a magnetic stirrer (Bibby Heated Magnetic Stirrer HB502, Sterilin, England) at room temperature to allow the complete dispersion of the Ch-Np. A concentration of 3% Ch-Np was selected for use in this study because of its ability to eradicate some endodontic pathogens in planktonic and biofilm states. 11 A mass of 1.6 g of Zeolite-Y (Zeolyst International, the Netherlands) was mixed with 3 mL of 3% (w/v) Ch-Np incrementally using a plastic spatula and a paper pad at room temperature for 30 seconds until a paste-like consistency was formed. This ratio was selected after validating a series of ratios that evaluated the consistency of the mixed material. The paste was then loaded into a 5-mL disposable plastic syringe for ease of use within the root canal system.

#### High-Resolution Scanning Electron Microscopy

The surface morphology of the synthesized Ch-Np powder and Zeolite-Y before and after mixing with Ch-Np was analyzed with high-resolution scanning electron microscopy (HR-SEM) using a Zeiss Gemini Auriga scanning electron microanalyzer equipped with a CDU-led detector at 3.00 kV with tungsten filament. A small amount of Ch-Np powder, Zeolite-Y, and Ch-Np-Zeolite nanocomposite was placed on stubs coated with carbon, and the samples were then coated with a thin gold film to make the surface conductive, prevent charging, and enhance the resolution of the images. HR-SEM images of Ch-Np powder and Zeolite-Y before and after mixing with Ch-Np were generated using different magnifications at various points of the samples.

#### Energy-Dispersive X-ray Spectroscopy Analysis

Quantitative elemental analysis and mapping using energy-dispersive X-ray spectroscopy (EDS) (field emission HR-SEM, Zeiss Gemini Auriga, Germany) were done for Zeolite-Y before and after mixing with Ch-Np to evaluate any changes in the elemental composition. The data were recorded for analysis.

### Antimicrobial Activity of Ch-Np-Zeolite Nanocomposite

#### Microbial Preparation

Following the characterization of the Ch-Np-Zeolite nanocomposite, its antimicrobial activity was assessed against two endodontic pathogens— *Streptococcus mutans* ( *S. mutans* ; ATCC 25175) (American Type Culture Collection, Manassas, Virginia, United States) and *Enterococcus faecalis* ( *E. faecalis* ; ATCC 29212)—in a planktonic state using an agar diffusion test. The effect of the presence of tissue inhibitors (dentine powder and serum albumin) was evaluated using the time-kill test performed via the broth microdilution technique.

Each microbial species was incubated for 24 hours in brain heart infusion (BHI) broth at 37°C before being subcultured on BHI agar plates and incubated for another 24 hours at 37°C. Cells from each microbial species were then suspended in phosphate buffer saline (PBS) solution to achieve a final concentration of 0.5 McFarland (McF) standard, which was adjusted using DensiCHEK Plus (BioMérieux, Durham, North Carolina, United States).

#### Agar Diffusion Test

Each microbial species was streaked onto poured BHI agar plates using a cotton swab. Five holes (5 mm in diameter) were made in each agar plate ( *n* = 15 for each species) and filled with the novel Ch-Np-Zeolite nanocomposite paste (experimental group [A] for *S. mutans* and experimental group [B] for *E. faecalis* ). The holes in the control groups were filled with Zeolite-Y mixed with distilled water (control group [C] for *S. mutans* and control group [D] for *E. faecalis* ). The agar plates were incubated for 24 hours at 37°C. After 24 hours, the zones of inhibition around each hole were measured in millimeters using an electronic digital caliper (Grip, RSA).

#### Effect of Tissue Inhibitors

The antimicrobial activity of the Ch-Np-Zeolite nanocomposite against *S. mutans* and *E. faecalis* was further tested in the presence of two tissue inhibitors (dentine powder and serum albumin) using a methodology adapted from Haapasalo et al 17 and Portenier et al. 18 Dentine powder was prepared from freshly extracted human teeth obtained from the Department of Oral Surgery, Faculty of Dentistry, University of the Western Cape. The teeth were extracted for orthodontic purposes. The teeth were kept in a 0.5% sodium hypochlorite solution (Milton, RSA) to prevent microbial growth and remove soft tissue. The coronal part of the teeth was removed with a sterile diamond bur on a high-speed handpiece. The pulp tissue was extirpated from all root canals using a barbed broach, and the roots were thoroughly rinsed with distilled water to remove any residual sodium hypochlorite. The roots were sterilized in an autoclave (Hirayama, Japan) at 121°C for 15 minutes before being crushed using a ball-milling machine (Fritsch Pulverisette, Germany) into a fine dentin powder.

The prepared dentine powder was suspended in 150 µL sterile BHI in Eppendorf tubes to form a final concentration of 56% (w/v). A volume of 150 µL Ch-Np-Zeolite nanocomposite was added to a 150-µL dentine powder suspension. Similar volumes were measured and mixed using serum albumin instead of dentine powder. The serum albumin was purchased from Thermo Scientific (South Africa) (Lot No.: RZB35918). Each mixture was allowed to mix for 2 hours before adding 150 µL of the previously prepared 0.5 McF standard of the tested microbial species.

Four groups were evaluated for each microbial species: two control groups containing either dentine powder (control group 1) or serum albumin (control group 2) without the Ch-Np-Zeolite nanocomposite and two experimental groups containing either dentine powder and Ch-Np-Zeolite nanocomposite (experimental group 1) or serum albumin and Ch-Np-Zeolite nanocomposite (experimental group 2). All groups were incubated under aerobic conditions in an orbital shaker incubator (Biocom Biotech, United States) at 37°C.

In a sterile 96-well microtiter plate, 50 µL from each group was added to a well that contained 50 µL of PBS. The suspension was diluted twofold by transferring 50 µL from the first well to the second one and then proceeding to the fifth one. The procedure of serial dilution was done at 0 minutes, 30 minutes, 1 hour, and 24 hours. A 2 µL of the final dilution from each group at each time was then transferred and streaked onto a BHI agar plate and incubated at 37°C for 24 hours under aerobic conditions. The number of colony-forming units (CFUs) in each plate was counted using an automated colony counter (Gerber, Lyss, Switzerland), following a standard protocol for the microbial counting of CFUs. 19 The test was repeated in triplicate (three independent experiments on separate days, each with three repeats for each organism), following the Clinical and Laboratory Standard Institute.

### Cytotoxicity Assay

The cytotoxicity assay was done by measuring the survival rate of the Balb/c 3T3 mouse fibroblast cell line (The National Repository for Biological Materials, Sandringham, Gauteng, South Africa) due to its ability to replicate without specific metabolic potential. 20 In addition, it is one of the most commonly used cell lines to evaluate the cytotoxicity of endodontic materials *in vitro* . 21 22 The cytotoxicity assay was done using the 3-(4,5-dimethylthiazol-2-yl)-2,5-diphenyltetrazolium bromide (MTT) colorimetric assay, as described by Mosmann. 23

The 3T3 Balb/c mouse fibroblast cells were grown to near confluency by adjusting their concentration to ∼3 × 10 ^5^ cells/mL, as per Grobler et al. 24 A volume of 100 µL of fibroblast cell suspension was plated in 96-well plates and allowed to attach to the well surface for 24 hours and reach a strong growth phase. The 3T3 fibroblast cells were then divided into three groups: a control group (1) containing 3T3 Balb/c mouse fibroblast cells and 100 µL Dulbecco's modified Eagles medium, an experimental group (2) containing Balb/c cells and 100 µL Zeolite-Y, and an experimental group (3) containing Balb/c cells and 100 µL Ch-Np-Zeolite nanocomposite. All groups were incubated for 24 hours at 37°C.

The survival rate of Balb/c 3T3 mouse fibroblast cells was evaluated using the MTT colorimetric assay. A mass of 5 mg of MTT (Merck, Sigma Aldrich, Saint Louis, Missouri, United States) was dissolved in 1 mL of PBS. A volume of 10 µL of the MTT was added to each well in each group, incubated at 37°C for 3 hours, and then discarded. The color change was then measured as represented by the optical density of the living cells when absorbed at a wavelength of 540 nm using a microplate reader (Rayto Rt–2100C, Shenzhen, China). The test was repeated three times (three independent experiments on separate days).

## Statistical Analysis

The antimicrobial and cytotoxic activities of the novel Ch-Np-Zeolite nanocomposite were analyzed using IBM SPSS statistics software (Version 25; IBM, Armonk, New York, United States). The difference between the antimicrobial activity of Ch-Np-Zeolite nanocomposite against *S. mutans* and *E. faecalis* was analyzed using the Mann–Whitney's *U* test. A probability value of *p* ≤ 0.05 was considered a significant difference. The effect of tissue inhibitors on the antimicrobial activity of Ch-Np-Zeolite nanocomposite against *S. mutans* and *E. faecalis* was analyzed by comparing the mean of log CFU/mL over time.

For the cytotoxicity assay, each group's mean optical density values were compared with the control group and expressed as a percentage of the control group, representing 100%. A statistically significant difference between each group and their control was made using a *t* -test with a probability value of *p* ≤ 0.05, considered to be a significant difference.

## Results

### Synthesis and Characterization of Ch-Np-Zeolite Nanocomposite

**Fig. 1:**
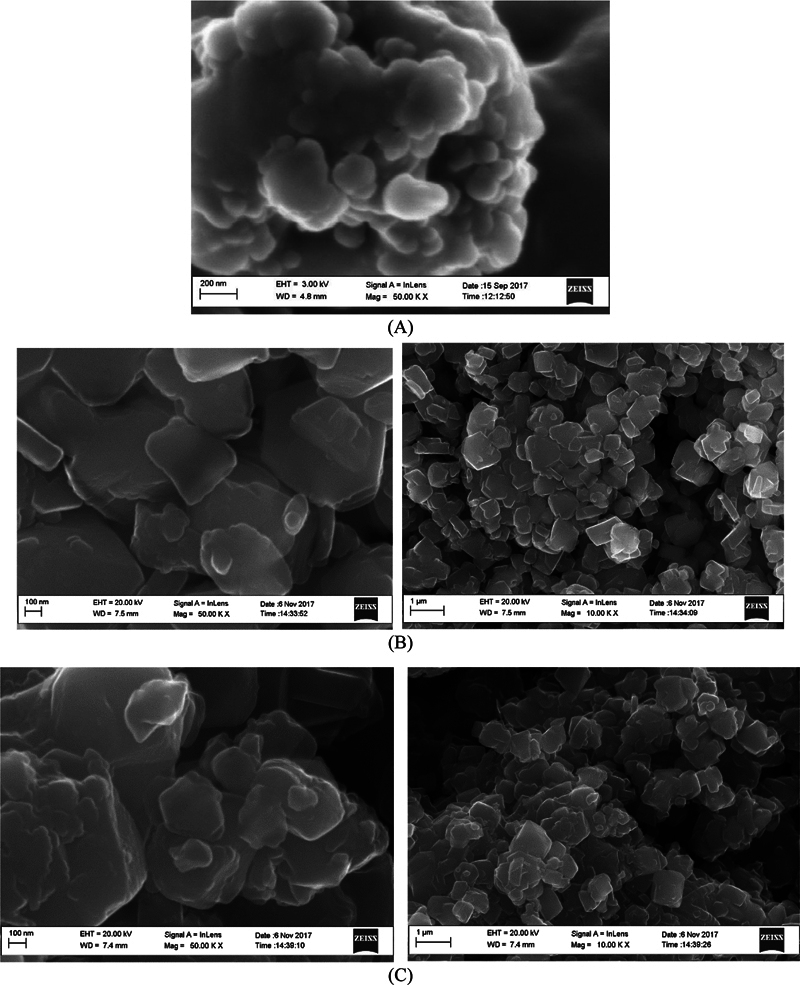
HR-SEM analysis of ( **A** ) Ch-Np powder, ( **B** ) Zeolite-Y before mixing with Ch-Np, and ( **C** ) Zeolite-Y after mixing with Ch-Np.

Mixing a colloidal suspension of Ch-Np with Zeolite-Y resulted in a white paste with a consistency suitable for placement as an intracanal medicament. The paste was loaded into a sterile disposable syringe for easy application. The HR-SEM analysis revealed that Ch-Np showed an irregular particle size in the range of 200 nm ( Fig. 1A ). Furthermore, the characteristic crystalline structure of Zeolite-Y did not alter following its mixing with the Ch-Np. HR-SEM showed the complete impregnation of the Ch-Np into the void volume of the microporous Zeolite-Y ( Fig. 1B, C ). EDS was used to determine the elemental composition of Zeolite-Y and Ch-Np-Zeolite nanocomposite. The Si:Al was 2.71 in Zeolite-Y, with a 2.4 atomic percentage of Na ^+^ . After mixing with Ch-Np, this ratio (Si:Al) was similar (2.69), and the atomic percentage of Na ^+^ was similar (2.58) following the mixture with Ch-Np ( Table 1 ).

**Table 1:** EDS analysis (elemental analysis) of zeolite-Y, and Ch-Np-Zeolite nanocomposite

Element	Atomic %	
	*Zeolite-Y*	*Ch-Np-Zeolite nanocomposite*
O	64.32	65.10
Na	2.4	2.58
Al	8.82	8.46
Si	23.97	22.84
Cl	0.52	0.93
Total	100	100

### Antimicrobial Effect of Ch-Np-Zeolite Nanocomposite

**Fig. 2:**
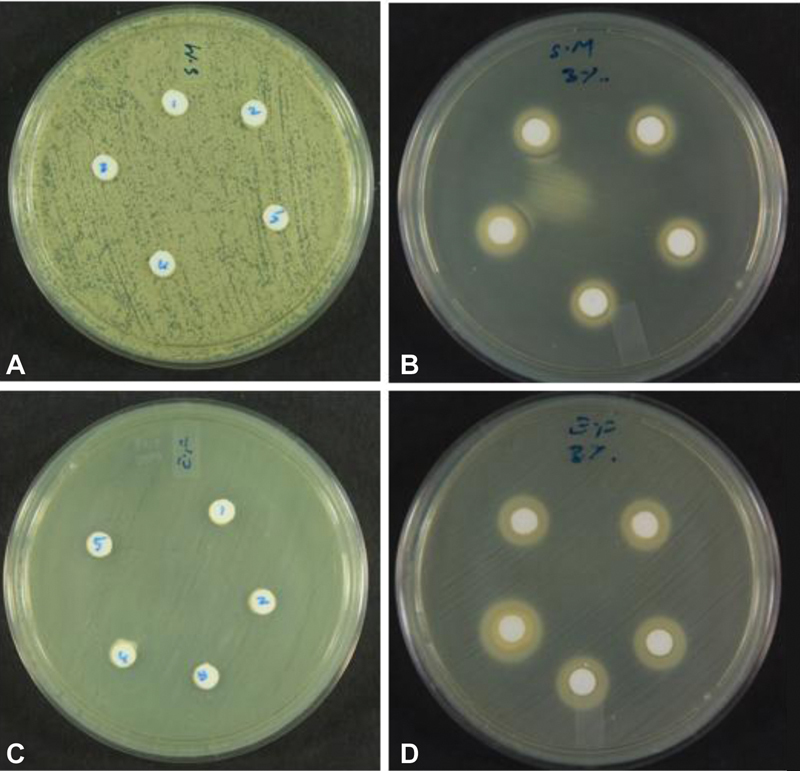
Brain-heart infusion agar plate of ( **A** ) Zeolite-Y only with S. *mutans* ; ( **B** ) Ch-Np-Zeolite nanocomposite with S. *mutans* ; ( **C** ) Zeolite-Y only with *E. faecalis* ; and ( **D** ) Ch-Np-Zeolite nanocomposite with *E. faecalis* .

**Fig. 3:**
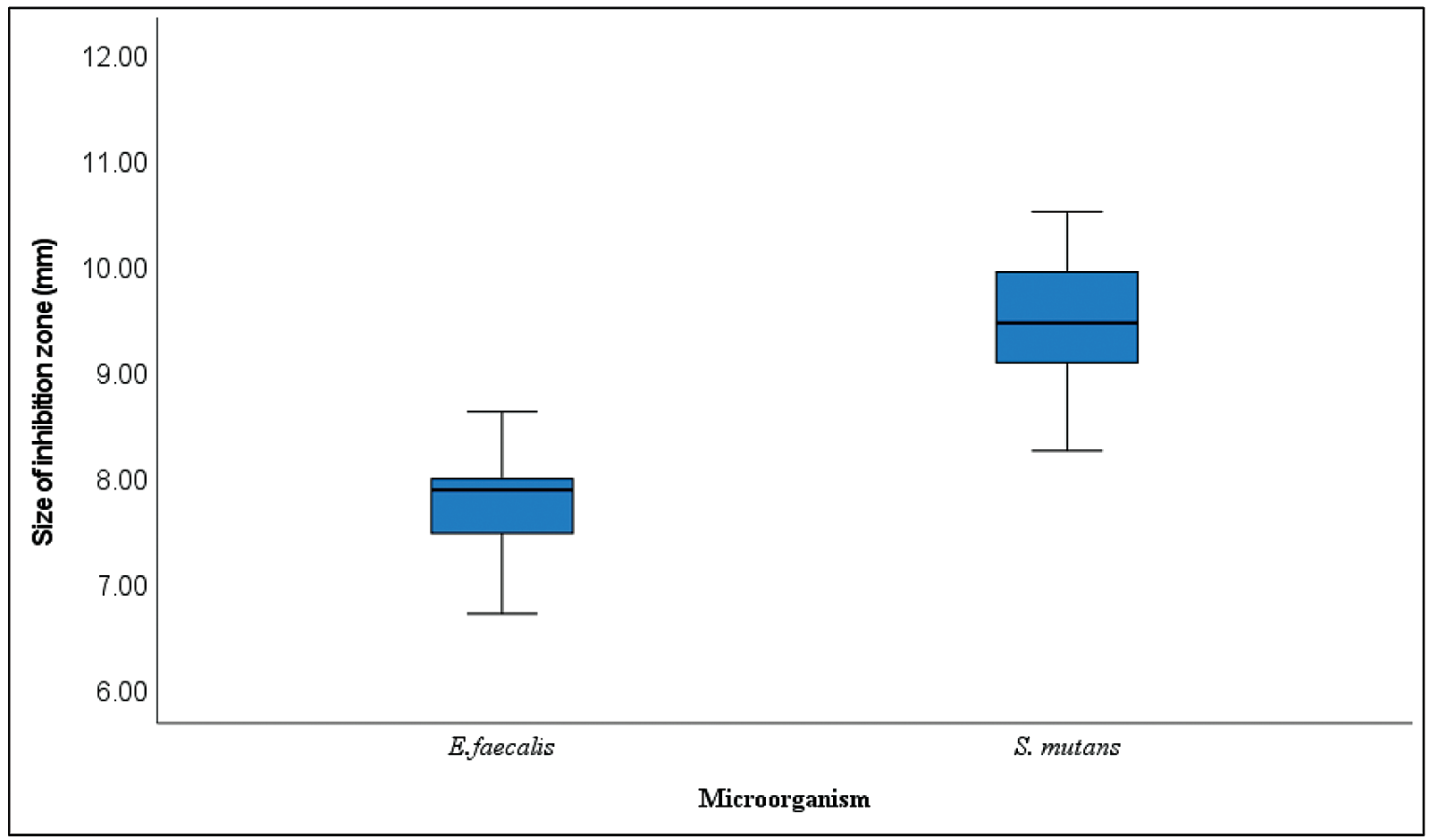
Box and Whisker plots showing the median, distribution, maximum, and minimum values of the size of the inhibition zone around *S. mutans* and *E. faecalis* produced by 3% Ch-Np-Zeolite nanocomposite.

The agar diffusion test showed no antimicrobial activity when using Zeolite-Y as a control against the two tested microbial species, indicating the lack of any antimicrobial effect against them by Zeolite-Y itself. The novel Ch-Np-Zeolite nanocomposite showed antimicrobial activity against *S. mutans* and *E. faecalis* , and a zone of inhibition was observed ( Fig. 2 ). The mean size of the inhibition zones when *S. mutans* and *E. faecalis* were exposed to the novel Ch-Np-Zeolite nanocomposite was 9.57 and 7.85 mm, respectively. The distribution of the size of the inhibition zones around *S. mutans* and *E. faecalis* was within the same range ( Fig. 3 ). A nonparametric Mann–Whitney's *U* -test was used to evaluate the difference in the antimicrobial activity of Ch-Np-Ze nanocomposite against *S. mutans* and *E. faecalis* . There was no significant difference in the efficacy of the nanocomposite against the two tested microbial species ( *p* = < 0.05), indicating equal effectiveness against both pathogens ( Table 2 ).

**Table 2:** Mann–Whitney's *U* test showing no statistical difference between the antimicrobial effect of Ch-Np-Zeolite nanocomposite against *S. mutans* and *E. faecalis*

Independent samples Mann–Whitney's *U* test	
Total	60
Mann–Whitney's *U* test	557.5
Standard error	63.273
Standardized test statistic	1.699
Asymptotic significance (two-sided test)	<0.05

#### Effect of Tissue Inhibitors on the Antimicrobial Activity of Ch-Np-Zeolite Nanocomposite

**Fig. 4:**
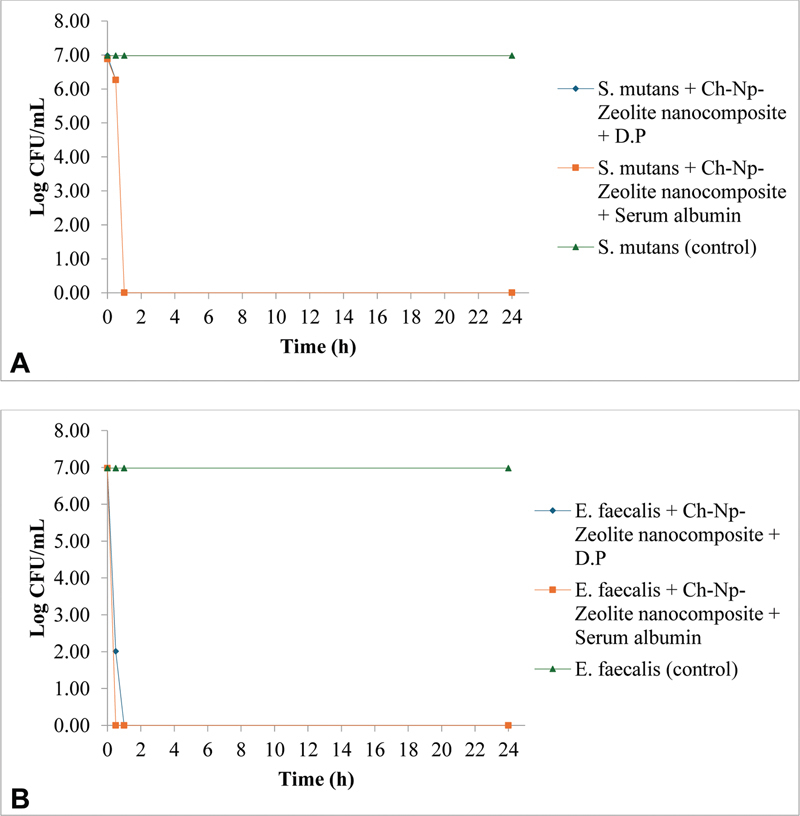
The distribution of the mean Log CFU/mL of ( **A** ) *S. mutans* when exposed to 3% Ch-Np-Zeolite nanocomposite in the presence of tissue inhibitors (dentine powder (D.P) and serum albumin); ( **B** ) *E. faecalis* when exposed to 3% Ch-Np-Zeolite nanocomposite in the presence of dentine powder (D.P) and serum albumin.

The antimicrobial activity of Ch-Np-Zeolite nanocomposite against *S. mutans* in the presence of dentine powder showed that at 0 minutes, the mean number of log CFU/mL was 6.98 for the control and experimental groups. However, this number declined to 6.27 at 30 minutes. It showed complete eradication at 1 hour when exposed to Ch-Np-Zeolite nanocomposite, compared with 6.98 CFU/mL at 30 minutes, which remained unchanged after 1 hour in the control group. The antimicrobial activity of the Ch-Np-Zeolite nanocomposite continued even after 24 hours of contact with *S. mutans* . In the presence of serum albumin, the number of CFU/mL of *S. mutans* at 0 minutes was 6.88 for both groups (control and experimental). This number reduced to 6.26 at 30 minutes and showed complete eradication at 1 hour, which continued until 24 hours of contact time ( Fig. 4A ).

The antimicrobial activity of Ch-Np-Zeolite nanocomposite against *E. faecalis* in the presence of dentine powder showed that at 0 minutes, the mean number of log CFU/mL of *E. faecalis* was 6.98 for both the control and experimental groups. This number declined rapidly to 2.01 at 30 minutes. It showed complete eradication of *E. faecalis* at 1 hour when exposed to Ch-Np-Zeolite nanocomposite, compared with 6.98 at 30 minutes, which remained unchanged after 1 hour in the control group. The antimicrobial activity of Ch-Np-Zeolite nanocomposite continued until the 24-hour contact in this study. In the presence of serum albumin, the number of CFU/mL of *E. faecalis* at 0 minutes was 6.98. This number declined rapidly to 0, showing complete eradication of *E. faecalis* at 30 minutes, which continued until tested 24-hour contact time ( Fig. 4B ).

The presence of tissue inhibitors (dentine powder and serum albumin) did not alter the novel Ch-Np-Zeolite nanocomposite's antimicrobial activity against *S. mutans* and *E. faecalis* when tested by measuring the number of their CFU/mL over time. Furthermore, the rapid decline in the log CFU/mL of *E. faecalis* indicates that the Ch-Np-Zeolite nanocomposite was more effective against *E. faecalis* than against *S. mutans* .

### Cytotoxicity Assay

**Fig. 5:**
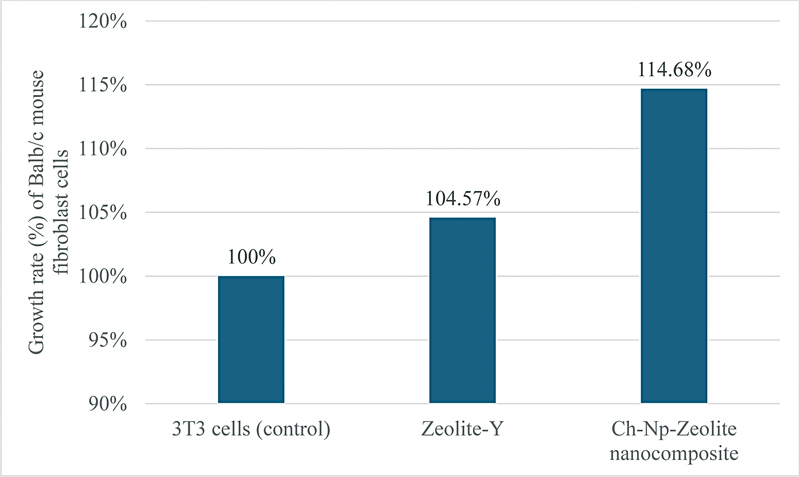
Bar graph showing the growth rate of Balb/c 3T3 mouse fibroblast cells when exposed to Zeolite-Y and Ch-Np-Zeolite nanocomposite.

Following exposure of Balb/c 3T3 mouse fibroblast cells to Zeolite-Y, the mean optical density values of the cells were 0.69 compared with their control (0.66). There was also a difference between the mean optical density values of the Balb/c 3T3 cells after their exposure to the novel Ch-Np-Zeolite nanocomposite and their control groups (0.58 and 0.51, respectively). Balb/c 3T3 mouse fibroblast cells showed a 100% growth rate in the control groups. Compared with the control values, the viable cells increased to 104.57 and 114.68% when exposed to Zeolite-Y and the novel Ch-Np-Zeolite nanocomposite, respectively ( Fig. 5 ), showing no inhibition and rather enhancing cell growth.

For further analysis, a comparison was made between the mean optical density values of each experimental group and their control group using a *t* -test to determine if there was any statistical difference. The growth rate of the Balb/c 3T3 cells following exposure to Zeolite-Y showed no statistical difference from that of their controls ( *p* < 0.05), indicating a similar growth rate in all groups. However, there was a statistical difference in the mean optical density of the Balb/c 3T3 cells after exposure to the novel Ch-Np-Zeolite nanocomposite compared with the control ( *p* < 0.05) ( Table 3 ), indicating an increase in its growth rate following its exposure to Ch-Np-Zeolite nanocomposite.

**Table 3:** *t* -test comparison of the growth rate of the Balb/c 3T3 mouse fibroblast cells following their exposure to Zeolite-Y and Ch-Np-Zeolite nanocomposite compared with their control groups using a *t* -test

Material	Group	Mean	Standard deviation	Mean difference	Standard error difference	95% confidence interval		*p* -Value
						Lower	Upper	
Zeolite-Y	Experimental group	0.69	0.08	0.03	0.02	0.00	0.06	< 0.05
	Control group	0.66	0.09					
Ch-Np-Zeolite nanocomposite	Experimental group	0.58	0.06	0.07	0.01	0.06	0.09	< 0.05
	Control group	0.51	0.04					

## Discussion

In this study, a composite prepared from nature-based Ch-Np and Zeolite-Y achieved high antimicrobial activity against endodontic pathogens. Mixing 3% (w/v) electrosprayed Ch-Np in water with Zeolite-Y powder at a concentration of 53.3 (w/v) resulted in a paste with a consistency suitable for use as an endodontic medicament. Furthermore, mixing Ch-Np with Zeolite-Y did not alter the morphological structure of Zeolite-Y when analyzed using HR-SEM; this may be due to the complete dispersion of Ch-Np in the water before mixing it with Zeolite-Y and the complete adsorption of Ch-Np into Zeolite-Y after mixing.

Zeolite-Y has a negatively charged surface due to the presence of negatively charged AlO _4_^−^ in their structure, which is charge-balanced by the presence of Na ^+^ . 25 The presence of Na ^+^ allows cation exchange and neutralization of the surface charge through the adsorption of Ch-Np. Furthermore, the interactions between the positively charged Ch-Np resulting from the electrospraying technique and the negatively charged Zeolite-Y surface may result in an electrostatic interaction that facilitates the adsorption of the Ch-Np onto the surface of Zeolite-Y. 26 Another possible mechanism that enables the adsorption of Ch-Np may be the binding of the amino group of Ch-Np to Zeolite-Y.

The antimicrobial activity of the Ch-Np-Zeolite nanocomposite was evaluated against two endodontic pathogens only as representatives of the primary endodontic infection pathogen ( *S. mutans* ) and persistent or secondary endodontic infection pathogens ( *E. faecalis* ). 27 28 The presence of an inhibition zone may result from the direct contact between the Ch-Np found on the surface of the nanocomposite and the tested microbial species or a continuous release of the Ch-Np from the Ch-Np-Zeolite nanocomposite structure.

Although the Ch-Np-Zeolite nanocomposite showed antimicrobial activity against the two tested pathogens, the antimicrobial activity was higher against *S. mutans* but not statistically different compared with *E. faecalis* . This may be due to the differences between *S. mutans* and *E. faecalis* in their structural and genetic mapping. 29 30 However, when tested in the presence of tissue inhibitors, *E. faecalis* showed a more rapid decline in the log CFU/mL compared with *S. mutans* .

One of the main findings of this study is the ability of the novel nanocomposite to maintain its antimicrobial activity against the tested pathogens in the presence of tissue inhibitors, which provides a superior advantage over traditional intracanal medicaments. This can be explained by the different mechanisms of action by the Ch-Np-Zeolite nanocomposite compared with the currently available intracanal medicaments, which can be ascribed to the ability of the Ch-Np to penetrate the microbial cell wall, disrupting its integrity and thus causing lysis. Furthermore, the highly positively charged Ch-Np that resulted from the electrospraying synthesis technique 11 may also facilitate its adhesion to the negatively charged microbial cell membrane and thus enhance antimicrobial activity. The potent antimicrobial activity of the Ch-Np-Zeolite nanocomposite, even in the presence of tissue inhibitors, may be due to the absence of any chemical interaction with the dentine structure or the serum albumin, which could alter its antimicrobial activity. Additionally, it has been shown that Ch-Np can chelate metallic ions inside the bacterial cell, which may further contribute to its antimicrobial mechanism. 31

Although the mechanism of increased cell growth of 3T3 fibroblast cells following their exposure to Ch-Np-Zeolite nanocomposite was not investigated, this enhancement may be due to the combination of Ch-Np with Zeolite-Y since neither Zeolite-Y nor electrosprayed Ch-Np showed any cytotoxic effect against the Balb/c 3T3 cell line. 11 The increase in Balb/c cell proliferation may allow the Ch-Np-Zeolite nanocomposite to promote healing of the injured periapical area if accidentally passed to the periapical tissue. However, further investigations are needed in this area.

This study has limitations, including evaluating only two types of endodontic pathogens. More research is needed to assess the antimicrobial activity of the synthesized Ch-Np-Zeolite nanocomposite against different endodontic pathogens in various states and compare its antimicrobial and cytotoxicity effects with currently available antimicrobial agents. Additionally, the extent of antimicrobial activity within the dentinal tubules over an extended period was not evaluated.

## Conclusion

Zeolite-Y can be used as a carrier to support well-dispersed nature-based antimicrobial Ch-Np, resulting in a nanocomposite suitable for use as a potential endodontic antimicrobial agent even in the presence of tissue inhibitors. However, more investigations are needed to assess its effectiveness against different endodontic pathogens and compare it to existing medicaments.
