# Supplementary figures and images for: Zeolite-Y-Loaded Chitosan Nanoparticles as Endodontic Antimicrobial Agent: An In vitro Study
Source: Eur J Dent. 2025 Apr 23;20(1):114–22. doi: 10.1055/s-0045-1802947 (PMC12890410; doi:10.1055/s-0045-1802947)

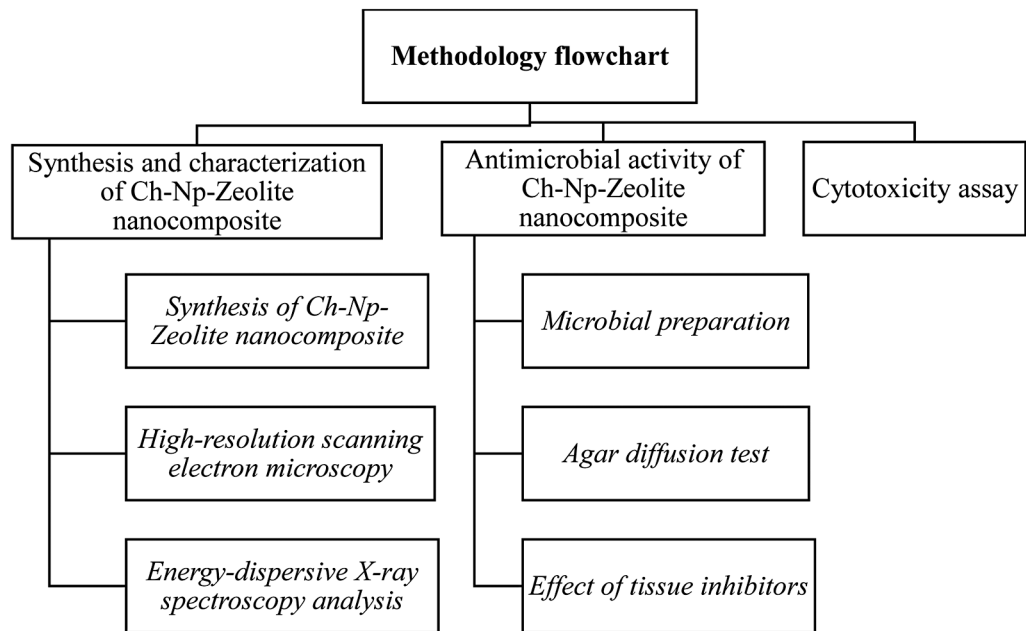

Supplementary Fig. S1

Supplement: Supplementary file 1 — Supplementary Material [file 10-1055-s-0045-1802947-s24103854.pdf]
